# PDZ binding kinase, regulated by FoxM1, enhances malignant phenotype via activation of β-Catenin signaling in hepatocellular carcinoma

**DOI:** 10.18632/oncotarget.17587

**Published:** 2017-05-03

**Authors:** Yu-Feng Yang, Ying-Hua Pan, Yun Cao, Jia Fu, Xia Yang, Mei-Fang Zhang, Qiu-Hong Tian

**Affiliations:** ^1^ Department of Pathology, Dongguan Third People's Hospital, Dongguan, China; ^2^ Department of Rheumatology and Immunology, The Third Affiliated Hospital of Sun Yat-Sen University, Guangzhou, China; ^3^ Department of Pathology, Sun Yat-Sen University Cancer Center, Guangzhou, China; ^4^ Department of Oncology, First Affiliated Hospital of Nanchang University, Nanchang, Jiangxi, China

**Keywords:** PBK, FoxM1, β-Catenin, HCC

## Abstract

Deregulation of serine/threonine kinase contributes to the development and progression of human diseases. PDZ-binding kinase (PBK) has been implicated in the malignant process of cancers, but its role and clinical significance in hepatocellular carcinoma (HCC) remains unclear. Here we show that PBK expression is increased and associated with larger tumor size, presence of vascular invasion, lymph node metastasis and poor overall and disease-free survivals in two independent cohorts of 879 patients with HCC. *In vitro* and *in vivo* data demonstrate PBK exerts oncogenic functions in HCC via activation of β-Catenin signaling pathway. The inhibition of β-Catenin by siRNAs or XAV-939 significantly attenuates PBK-mediated malignant phenotypes. PBK is further identified as a downstream effector of FoxM1. In clinical samples, PBK expression is positively correlated with the expression of FoxM1 and nuclear β-Catenin. Collectively, these findings suggest PBK functions as an oncogene in HCC and the newly identified FoxM1/PBK/β-Catenin axis serves as a promising prognostic factor as well as therapeutic intervention for HCC.

## INTRODUCTION

Efforts have been made to deal with the high mortality of hepatocellular carcinoma (HCC) that ranks top three of the cancer-related death last decades [1]. Outcomes remains extremely poor for patients with HCC, despite of the improved strategies in disease diagnosis and treatment [2]. The encouraging efficacy of immunological and targeted therapies in other cancer types may bring new hope for the clinical management of HCC [3, 4]. The priority of precision medicine is the identification of biomarkers that are detectable and drugable. As a result, it is of essential significance to search for the proteins benefiting the malignant progress of human cancers.

Deregulation of protein kinases involved in p38 mitogen-activated protein kinase (MAPK) pathway is response for the uncontrolled tumor growth and metastasis [5, 6]. PDZ-binding kinase (PBK), a serine/threonine kinase, functions as the upstream kinase of p38/ERK signaling [7]. PBK is rarely found in normal tissues except the fetal and germ cells. Its increased expression during mitosis suggest a role of PBK in cell growth [8]. Accumulating literatures provide evidence that PBK is deregulated and plays an important role in human cancers. Park et al. showed that PBK was up-regulated in breast cancer and exerted oncogenic activity [9]. PBK was found to interact with p53, and subsequently suppress the expression of p21 to accelerate the cell cycle in colorectal carcinoma cells [10]. In lung cancer, PBK promoted the phosphorylation of AKT, but decreased the expression of PTEN to facilitate the cell migration and invasion [11]. On the other hand, more and more attentions have been paid to unveil the clinical implication of PBK. The overexpression of PBK was revealed as a unfavorable factor for overall survival of patients with lung cancer [12], gastric cancer [13] and nasopharyngeal carcinoma [14]. However, the role of PBK and its clinical value in HCC remains unclear.

Using tissue microarray (TMA)-based immunohistochemistry on two independent cohorts of 879 patients with HCC, *in vitro* experiments and mice models, we intended to disclose the relevant clinical significance of PBK, to investigate the biological function and the underlying mechanism of PBK in the progression of HCC. Our data identify PBK as an oncogene in HCC and suggest PBK as a potential prognostic and therapeutic biomarker in this deadly disease.

## RESULTS

### PBK is up-regulated in HCC and associated with poor outcomes

**Figure 1:**
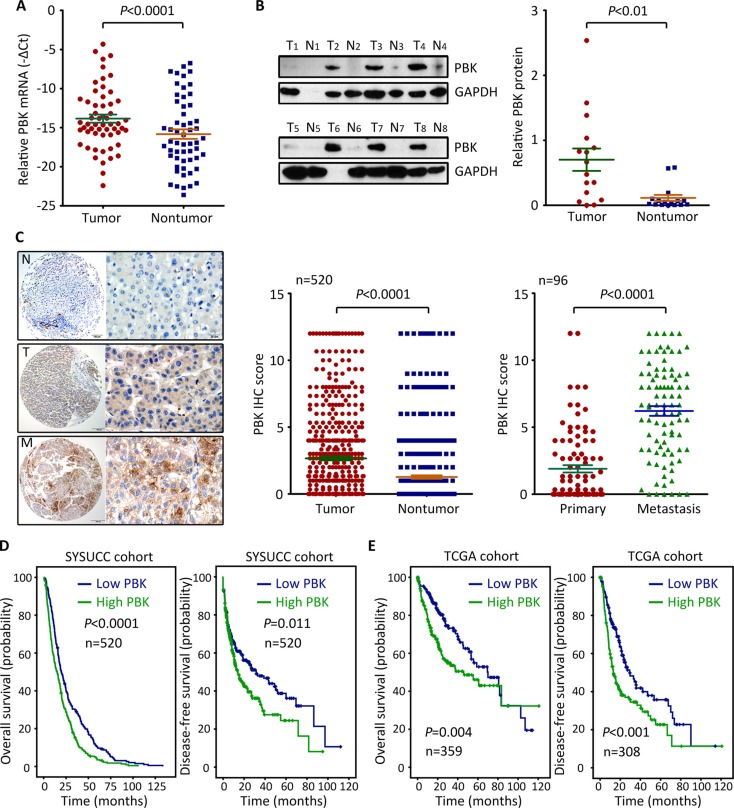
PBK expression is up-regulated in HCC and associated with poor outcomes (**A**) The expression of PBK mRNA in 56 pairs of HCC specimens was determined by qRT-PCR. (**B**) PBK protein level was evaluated in 16 HCC cases by western blot. The representative images (left panel) and the statistics (right panel) were presented. (**C**) PBK expression was examined 520 paired HCC and nontumorous tissues and 96 paired HCC and metastatic tissues by immunohistochemistry (IHC). The IHC score of PBK was shown and compared. (**D**, **E**) Kaplan-Meier analyses was conducted to assess the value of PBK in overall and disease-free survivals of patients with HCC in our and TCGA cohorts.

To determine the expression of PBK mRNA in HCC, 56 fresh specimens were collected for qRT-PCR. The mRNA level of PBK in HCC tissues were significantly higher than those in nontumorous tissues (Figure 1A). Consistently, PBK protein expression was noticeably increased in HCC tissues, compared with the corresponding adjacent liver tissues where PBK protein was hardly detected (Figure 1B). To further validate the up-regulation of PBK in HCC, a cohort of 520 patients with HCC was recruited. TMA-based IHC showed positive staining of PBK in 77.3% (402/520) of HCC tissues, but only in 15.2% (79/520) of nontumorous tissues (Figure 1C). Furthermore, more expression of PBK in the portal vein embolus was found in 78.1% (75/96) of cases, compared with the primary tumor (Figure 1C).

According to the median score of PBK IHC, patients were divided into two groups: high PBK expression and low PBK expression. High PBK expression was significantly associated with larger tumor size (*P* = 0.001), vascular invasion (*P* = 0.003) and tumor lymph node metastasis (*P* = 0.019) (Table 1). Patients with low PBK expression in our cohort were likely accompanied with a longer overall and disease-free survival, according to the Kaplan-Meier analysis (Figure 1D). Multivariate analysis revealed that PBK expression was an independent predictor for overall survival (hazard ratio = 1.411, 95% confident interval: 1.171–1.701, *P* < 0.001) (Table 2), but not for disease-free survival (data not shown). The prognostic implication of PBK in HCC was confirmed in TCGA cohort. Patients with high PBK mRNA expression survived much shorter and experienced short period of tumor relapse or metastasis (Figure 1E). Collectively, these data imply that PBK is overexpressed in HCC and of clinical significance in patient prognosis.

**Table 1:** Correlation of clinicopathological features and PBK expression

Variable	PBK expression			
	All cases	Low expression	High expression	*P* value^a^
Age (years)^b^				0.830
< 49	239	106 (44.4%)	133 (55.6%)	
≥ 49	281	122 (43.4%)	159 (56.6%)	
Gender				**0.003**
Male	470	196 (41.7%)	274 (58.3%)	
Female	50	32 (64.0 %)	18 (36.0%)	
HBsAg				0.214
Positive	80	30 (37.5%)	50 (62.5%)	
Negative	440	198 (45.0%)	242 (55.0%)	
AFP (ng/ml)				0.523
< 20	123	57 (46.3%)	66 (53.7%)	
≥ 20	397	171 (43.1%)	226 (56.9%)	
Cirrhosis				0.326
Yes	433	194 (44.8%)	239 (55.2%)	
No	87	34 (39.1%)	53 (60.9%)	
Tumor size (cm)				**0.001**
< 5	112	64 (57.1%)	48 (42.9%)	
≥ 5	408	164 (40.2%)	224 (59.8%)	
Tumor multiplicity				0.355
Single	326	148 (45.4%)	178 (54.6%)	
Multiple	194	80 (41.2%)	114 (58.8%)	
Tumor Differentiation				0.535
Well-Moderate	376	168 (44.7%)	208 (55.3%)	
Poor-undifferentiated	144	60 (41.7%)	84 (58.3%)	
TNM				0.107
I–II	278	131 (47.1%)	147 (52.9%)	
III–IV	242	97 (40.1%)	145 (59.9%)	
Vascular invasion				**0.003**
Yes	111	35 (47.2%)	76(52.8%)	
No	409	193 (31.5%)	216 (68.5%)	
Tumor Capsule				0.302
Absent	296	124 (41.9%)	172 (58.1%)	
Present	224	104 (46.4%)	120 (53.6%)	
LNM				**0.019**
No	487	220 (45.2%)	267 (54.8)	
Yes	33	8 (24.7%)	25 (75.8%)	

^a^Chi-square test; ^b^Median age; AFP, alpha-fetoprotein; HBV, hepatitis B virus; LNM, lymph node metastasis.

**Table 2:** Univariate and multivariate analyses of PBK expression and overall survival

Variables	Univariate analysis		Multivariate analysis	
	HR (95% CI)	*P* value	HR (95% CI)	*P* value
Age (< 49 vs. ≥ 49 years)	0.868 (0.728–1.035)	0.114		
Gender (female vs. male)	1.021 (0.758–1.376)	0.891		
HBV (positive vs. negative)	1.092(0.857–1.391)	0.478		
Tumor size (< 5 vs. ≥ 5 cm)	1.420(1.148–1.757)	**0.001**	1.143 (1.896–1.459)	0.281
Tumor multiplicity (single vs. multiple)	1.199 (1.000–1.436)	**0.050**	0.961(0.779–1.184)	0.701
Tumor capsule (absent vs. present)	0.715 (0.597–0.852)	**0.000**	0.854(0.708–1.031)	0.100
Liver cirrhosis (yes vs. no)	0.744 (0.588–0.941)	**0.013**	0.867 (0.676–1.111)	0.260
AFP (< 20 vs. ≥ 20 ng/mL)	1.407 (1.145–1.728)	**0.001**	1.240 (1.003–1.533)	**0.047**
Vascular invasion (yes vs. no)	1.866 (1.504–2.316)	**0.000**	1.322(1.034–1.690)	**0.026**
Tumor differentiation	1.454 (1.195–1.768)	**0.000**	1.284 (1.051–1.569)	**0.015**
TNM (I–II vs. III–IV)	1.555 (1.303–1.855)	**0.000**	1.284 (1.009–1.635)	**0.042**
LNM (yes vs. no)	1.568 (1.100–2.335)	**0.013**	1.343 (0.926–1.948)	0.120
PBK expression (low vs. high)	1.517 (1.269–1.814)	**0.000**	1.411 (1.171–1.701)	**0.000**

AFP, α-fetoprotein; HBsAg, hepatitis B surface antigen; HR, hazard ratio; CI, confidence interval; LNM, lymph node metastasis.

### PBK exhibits oncogenic activities in HCC

**Figure 2:**
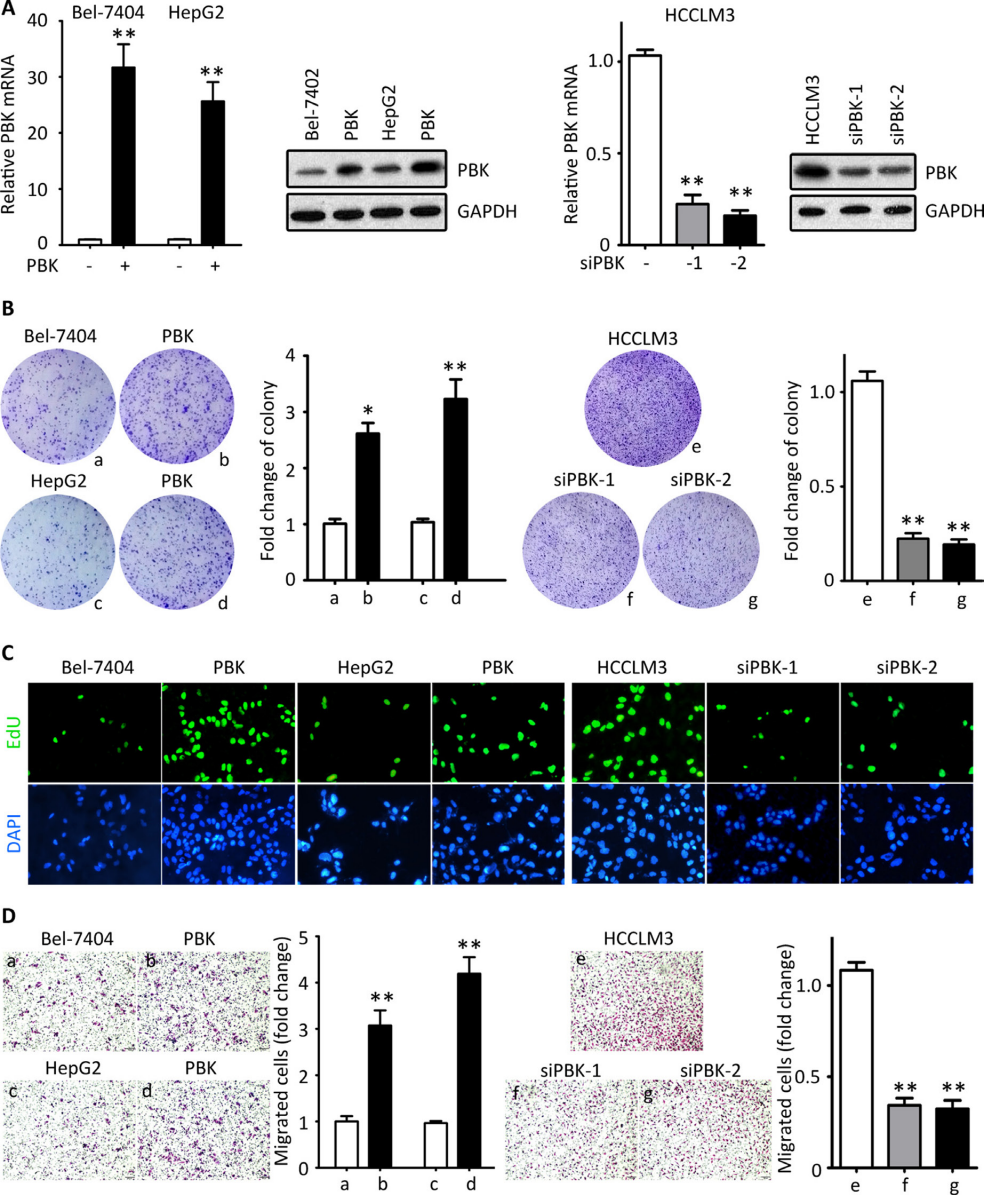
PBK promotes HCC cell proliferation and migration *in vitro* (**A**) PBK mRNA and protein expressions were determined in Bel-7404 and HepG2 cells transfected with either PBK plasmid for 36 h or PBK siRNAs for 36 h. (**B**) Colony formations assays were performed, using cells with PBK overexpression or knockdown. (**C**) The cell proliferation was determined by EdU staining. (**D**) Transwell assays were used to assess the effect of PBK on cell migration. **P < 0.01.

Since the analyses of clinical correlation indicate PBK might be involved in tumor progression, we performed *in vitro* and *in vivo* experiments to disclose the role of PBK in HCC. PBK expression was either induced in Bel-7404 and HepG2 cells or knocked down in HCCLM3 cells (Figure 2A). Colony formation and EdU staining were used to evaluate the effect of PBK in HCC cell proliferation. Ectopic expression of PBK marked enhanced the ability of colony formation, whereas the PBK-silenced cells failed to form foci (Figure 2B). Results of EdU staining showed that cells under proliferation were more often depicted in cells with PBK expression. In contrast, PBK siRNAs were dramatically reduced the EdU-positive HCCLM3 cells (Figure 2C). These findings indicated that PBK was capable of promoting cell growth. On the other hand, Transwell assays were utilized to assess the impact of PBK in cell migration. Exogenous PBK expression increased the cell migration, whereas PBK depletion attenuated the cell movement (Figure 2D).

**Figure 3:**
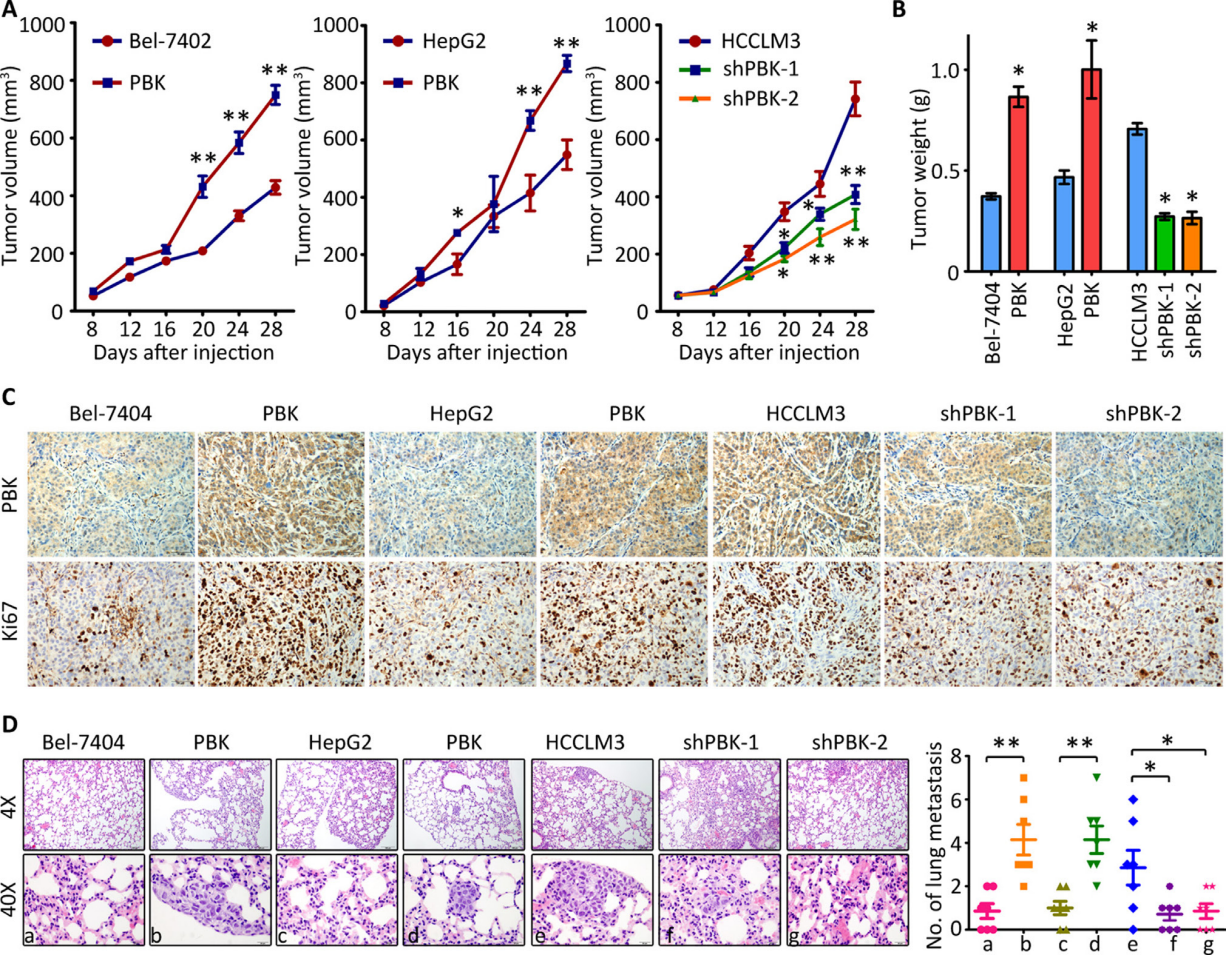
PBK enhances tumor growth and metastasis *in vivo* (**A**) HCC cells were injected into the left flank of the mice. The tumor volumes were measured every 3 days. The growth curve was recorded. (**B**) At day 28 after injection, tumors were resected and weighted. (**C**) The paraffin-embedded tumors were sectioned and stained with PBK and Ki67. (**D**) Cells were injected into the tail vein. After 40 days, the lungs were dissected and stained with HE. The metastatic nodules were counted under a microscope and indicated. All *P < 0.05, **P < 0.01.

We next investigated whether PBK affected the tumor growth and metastasis *in vivo*. For Bel-7404, tumors were found in 6/6 and 4/6 of null mice in overexpression group and control group, respectively. For HepG2, the relevant numbers were 6/6 and 5/6, respectively. For HCCLM3, tumors were found in 3/6, 3/6 and 6/6 of null mice in knockdown groups and control group, respectively. The tumors in PBK groups grew more faster than those in control group. Conversely, tumors expressing less PBK experienced growth suppression (Figure 3A). Compared to the control groups, the tumors were much heavier in PBK overexpression group but much lighter in PBK depletion group (Figure 3B). IHC was performed to confirmed the alteration of PBK expression in each group. PBK-expressing cells were associated with more expression of Ki67, a biomarker for evaluation of cell proliferation (Figure 3C). Caudal vein injection model was used to assess the effect of PBK on tumor metastasis. Hematoxylin-eosin (H&E) staining revealed the lung metastasis was more often depicted in PBK overexpression groups, compared to the control groups. Statistically, there was altered ability of the cells to metastasize to the lung (Figure 3D). Collectively, these findings indicate that PBK contributes to the HCC progression.

### PBK triggers β-catenin signaling pathway to promote HCC

**Figure 4:**
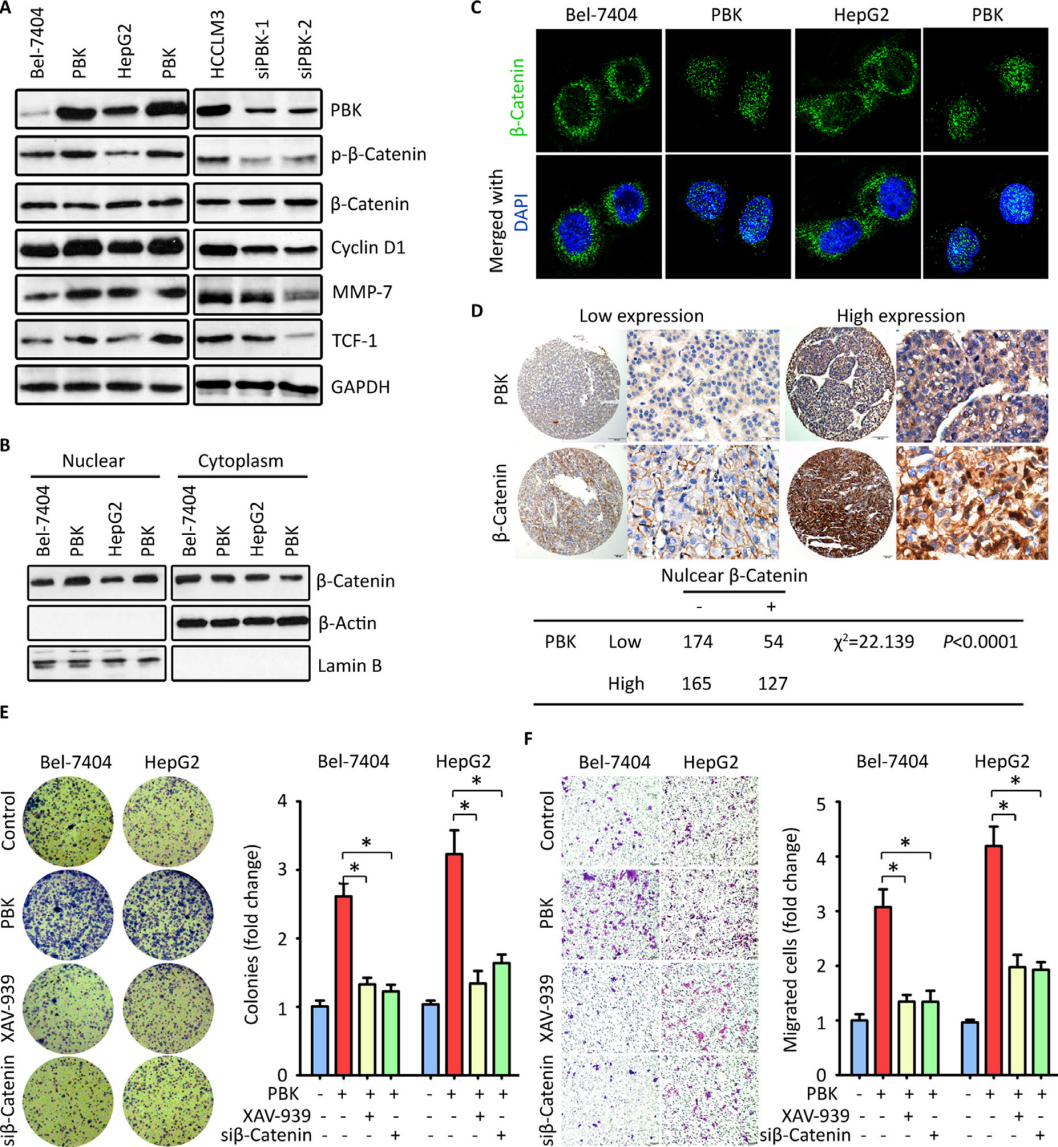
PBK exerts oncogenic activities by activating β-Catenin signaling pathway (**A**) HCC cells were transfected with PBK overexpression vector or siRNAs for 36 h. The expressions of phophorylated-β-Catenin, Cyclin D1, MMP-7 and TCF-1 were examined by western blot. GAPDH was served as loading control. (**B**) The cells treated as described in A were fractioned into nuclear and cytoplasm. The level of β-Catenin was determined. (**C**) Immunofluoresence was performed to indicate the cellular localization of β-Catenin in cells with PBK overexpression. (**D**) The representative images of PBK and β-Catenin IHC staining were presented (top panel). The correlation between PBK and β-Catenin expression in HCC tissues was determined and shown (bottom panel). (**E**, **F**). Cells with CBX8 overexpression were treated with either β-Catenin siRNA or its inhibitor XAV-939. Colony formation (E) and Transwell assays (F) were performed to evaluate the effect of inhibition of β-Catenin on the CBX8-promoted cell proliferation and migration. The fold changes were indicated by histogram. All *P < 0.05.

To uncover the underlying mechanism of PBK-mediated oncogenic activity, we examined the effect of PBK on the activation of β-catenin signaling. Western blot showed that the phosphorylation of β-catenin at Ser552 was enhanced by PBK reintroduction in Bel-7404 and HepG2 cells, but inhibited by PBK siRNAs in HCCLM3 cells. The expressions of β-catenin-regulated targets, including MMP-7, cyclin D1 and TCF-1, were accordingly altered by PBK expression (Figure 4A). Fraction experiments showed that the nuclear β-catenin was increased by PBK overexpression, but decreased in HCC cells with PBK knockdown (Figure 4B). IF staining presented the nuclear localization of β-catenin in cells with PBK transfection (Figure 4C). The PBK-mediated β-catenin translocation to the nuclear was supported by the clinical data. In HCC tissues, high PBK expression was correlated with the presence of nuclear β-catenin in 520 cases (Figure 4D).

The profound impact of PBK on activation of β-Catenin signaling prompted us to examine the effect of β-Catenin inhibition on PBK-mediated malignant activities. Cells with PBK overexpression were treated with XAV-939 (an inhibitor for β-Catenin) or β-Catenin siRNA. Colony formation and Transwell assays were performed. Results demonstrated that PBK-promoted hepaotosphere formation was abolished by XAV-939 and β-Catenin siRNA (Figure 4E). Similarly, the enhanced cell migration by PBK overexpression was partly rescued (Figure 4F). Taken together, our data indicate that PBK exhibits proHCC activity via the activation of β-Catenin pathway.

### PBK is transrciptionally regulated by FoxM1

**Figure 5:**
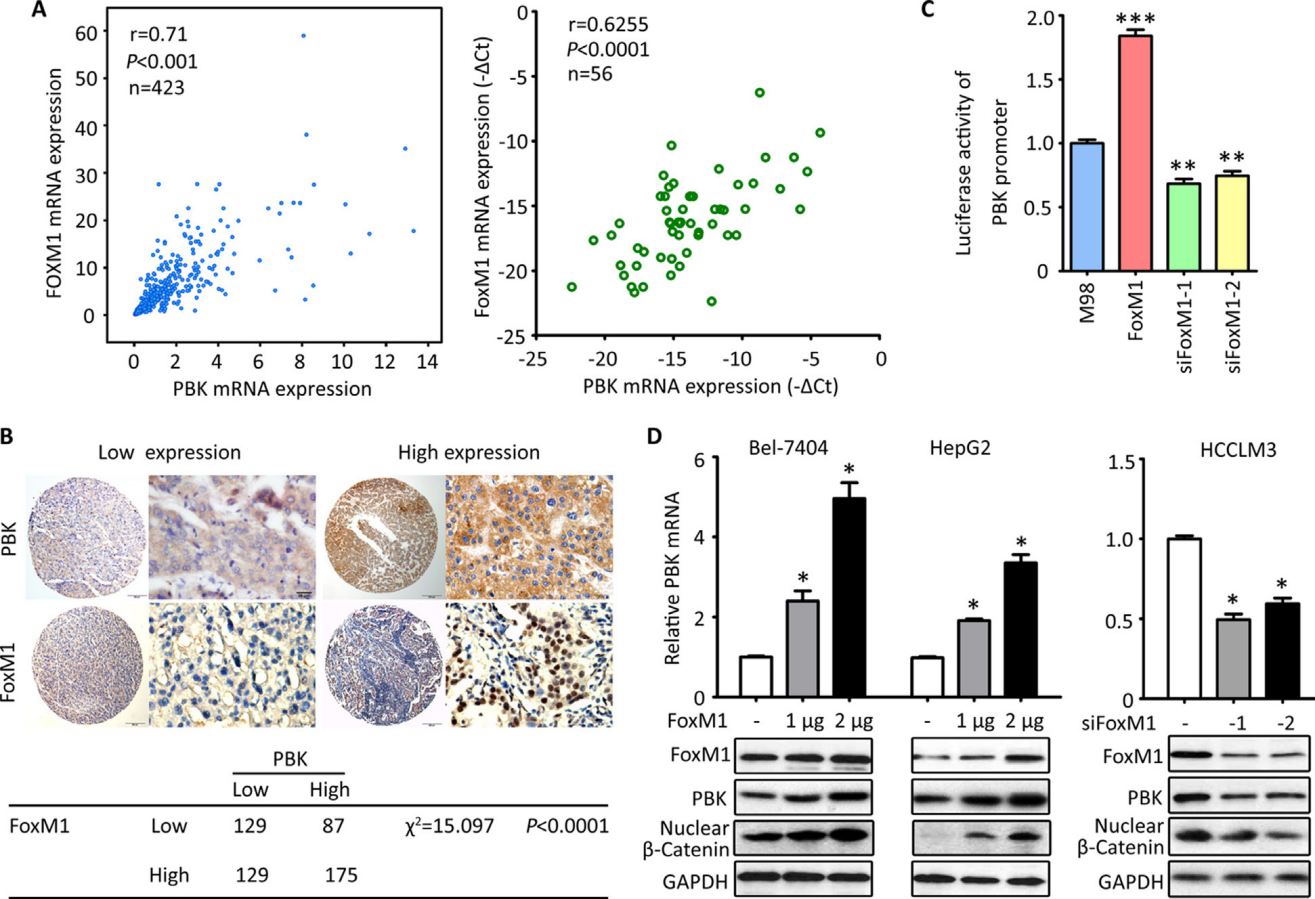
PBK is transcriptionally regulated by FoxM1 (**A**) The correlation of PBK and FoxM1 mRNA expression was determined in 423 cases in TCGA cohort (left panel) and 56 cases in our cohort (right panel). (**B**) The representative images of PBK and FoxM1 IHC staining were presented (top panel). The correlation between PBK and FoxM1 expression in 520 HCC tissues was determined and shown (bottom panel). (**C**) Luciferase assays were performed to effect of FoxM1 overexpression or knockdown on the activity of PBK promoter in Bel-7404 cells. (**D**) The effect of FoxM1 overexpression or knockdown on PBK expression was determined in HCC cells. All *P < 0.05, **P < 0.01.

Based on the finding that PBK was rarely found in normal tissues but markedly up-regulated in HCC, we next intended to find out the reasons. According to the TCGA data, PBK mRNA expression was positively coexpressed with the mRNA expression of FoxM1 in 423 patients (Figure 5A). In 56 HCC cases, the PBK mRNA was closely correlated with FoxM1 mRNA (Figure 5A). Furthermore, HCC cases with high expression of PBK protein was frequently with high expression of FoxM1 in our cohort of 520 patients (Figure 5B). These connections may imply the regulation of PBK by FoxM1. The luciferase reporter assay confirmed that FoxM1 was capable of modulating the activity of PBK promoter, showing that the related luciferase activity of PBK promoter was enhanced by PBK overexpression but attenuated by PBK depletion in Bel-7404 cells (Figure 5C). In HCC cells, FoxM1 up-regulated the expression of PBK and β-Catenin at both mRNA and protein levels in a dose-dependent manner. In consistent, the knockdown of FoxM1 led to the decrease the expression of PBK and nuclear β-Catenin (Figure 5D). These findings implicate that FoxM1 serves as a up-stream regulator of PBK in HCC cells.

## DISCUSSION

Searching for biomarkers useful for diagnosis and personalized therapy has been attracting more and more interests. During the last decades, efforts have been made to disclose the mechanism of the development and progression of HCC, aiming to explore new strategy for the clinical management of this deadly disease. Advances in molecular biology have recently led to the rapid development of individualized cancer management. Here, we identify PBK, a downstream effector of FoxM1, as an oncogene in HCC via the activation of β-Catenin signaling pathway. Clinical data reveal the expression of PBK was of prognostic value in HCC to provide promising hints for clinical management.

Deregulation of PBK has been reported in other cancer types. Ohashi et al. reported that PBK was up-regulated in esophageal squamous cell carcinoma and associated with worse prognosis [16]. Kwon et al. showed that PBK expression in gastric carcinoma was significantly increased and predicted poor overall survival [17]. Chen and colleagues provided evidence that PBK was induced in prostate cancer and connected with high rate of tumor recurrence [18]. In our study, PBK expression in HCC was gradually increased from nontumorous, primary to metastatic tumor. Cohort studies indicate that high PBK expression was correlated with unfavorable overall and disease-free survival in a total of 879 patients. Collectively, our data complement the clinical significance of PBK in HCC.

Due to the specific expression in cancer cells, targeting PBK has been attempted as a potential therapy for HCC. Treated with HITOPK-032 (an inhibitor for PBK), subcutaneous tumors were strikingly diminished in mice models of colon cancer [19], glioma [20] and nasopharyngeal carcinoma [14]. In a latest study, Song and colleagues treated liver cancer cells with Glycycoumarin that inactivates PBK, and observed tumor reduction [21]. Although there are still lots of pre-clinic researches to confirm the feasibility of PBK as a therapeutic target, the data are encouraging due to the limited progress of HCC treatment. Our data demonstrated that PBK promoted tumor growth and metastasis via activating β-Catenin signaling pathway which plenty of chemotherapeutic agents target. Whether the combination of anti-PBK and anti-β-Catenin exerts synergistic effect in HCC requires further investigation.

Aberrant activation of β-Catenin signaling contributes to the initiation and progression of HCC. Our data demonstrated that overexpression of PBK resulted in the phosphorylation and the accumulation of nuclear β-Catenin, indicating that PBK manifests its protumor activity by enhancing the transactivation of β-Catenin. As the upstream of MAPK pathway, PBK triggers the activation of p38 and ERK. The cross-talk between MAPK and β-Catenin pathway has been reported previously. Thornton et al. showed that phosphorylation of MAPK led to the accumulation of nuclear β-Catenin via inactivation of GSK-3β [22]. Gu et al. demonstrated that both MAPK and β-Catenin pathways were activated by the loss of tumor suppressor candidate 3 (TUSC3) [23]. These data might suggest that once PBK is suppressed, two of the important pathways in cancer could be blocked.

Our data showed that PBK was a direct target of FoxM1, one of the famous transcription factor (TF) [24, 25]. The overexpression of PBK in human cancers is attributed to the deregulation of TFs, such as c-Myc [26], E2F and CREB/ATF [27]. Interestingly, E2F was required in p53-mediated FoxM1 regulation [28]. c-Myc and FoxM1 were co-operated in Ras-driven hepatocarcinogenesis [29]. These data might imply a network of c-Myc, E2F and FoxM1 in the regulation of PBK expression. Taken together, our study provide convincing evidences that FoxM1-regulated PBK exerts oncogenic activities towards HCC via the activation of β-Catenin pathway. The newly identified FoxM1/PBK/β-Catenin axis serves as promising prognostic factor and therapeutic intervention for HCC.

## MATERIALS AND METHODS

### Patients and specimens

Five hundred and twenty HCC cases diagnosed between Jan 2011 to Dec 2011 at Dongguan Third People's Hospital and First Affiliated Hospital of NanChang University, were recruited in this study. Another 56 fresh specimens were collected for qRT-PCR and western blot. Ninety six HCC cases, along with the venous metastases, were obtained. None of the patients had received radiotherapy or chemotherapy before surgery. This project was approved by Institute Research Ethics Committee of the First Affiliated Hospital of NanChang University. All samples were anonymous. The prognostic value of PBK was further validated in a cohort of The Cancer Genome Atlas (TCGA) dataset (http://www.cbioportal.org).

### Cell culture and transfection

Bel-7404, HepG2 and HCCLM3 cells were purchased from the Cell Resource Center, Chinese Academy of Science Committee (Shanghai, China), and maintained in Dulbecco's modified Eagle's medium (DMEM) (Gibco, Gaithersburg, MD, USA) supplemented with 10% heat-inactivated fetal bovine serum (FBS, Hyclone, Logan, UT) in a humidified incubator at 37°C and 5% CO_2_. The cells were transfected with PBK overexpression vector and siRNAs with Lipofectamine 2000, according to the instruction.

### Quantitative real-time polymerase chain reaction (qRT-PCR)

Total RNA was extracted using the Trizol Reagent (Invitrogen, Carlsbad, CA, USA). cDNA was synthesised using Moloney murine leukaemia virus reverse transcriptase (Promega, Madison, WI, USA). RT-PCR was carried out with the following cycling conditions: 95°C for 10 min, 40 cycles of 94°C for 30 s, 60°C for 30 s, 72°C for 30 s and a final extension of 10 min at 72°C. The sequences of the PCR primers are as following: PBK, forward: 5′-GAAGAGGACTGAGAGTGGCT-3′ and reverse: 5′-CTTCTGCATAAACGGAGAGGC-3′; FoxM1, forward: 5′-GAGACCTGTGATGGTGAGGC-3′ and reverse: 5′- ACCTTAACCTGTCGCTGCTC-3′; β-actin, forward: 5′-TGGCACCCAGCACAATGAA-3′ and reverse: 5′-CTAAGTCATAGTCCGCCTAGAAGCA-3′.

### Western blot

Western blot was performed as described in our previous study [15]. The primary antibodies were as followings: PBK (1:1000, #4942, Cell Signaling Technology), FoxM1 (1:1000, #5436, Cell Signaling Technology), β-catenin (1:1000, #9582s, Cell Signaling Technology), p-β-catenin (1:1000, # 5651s, Cell Signaling Technology) GAPDH (1:1000, Santa Cruz).

### Immunohistochemistry (IHC)

TMA sections with a thickness of 4 μm were dewaxed in xylene and graded alcohols, hydrated, and washed in phosphatebuffered saline (PBS). After pretreatment in a microwave oven, endogenous peroxidase was inhibited by 3% hydrogen peroxide in methanol for 20 min, followed by avidin-biotin blocking using a biotin-blocking kit (DAKO, Germany). Slides were then incubated with primary antibodies, overnight in a moist chamber at 4°C, washed in PBS, and incubated with biotinylated goat anti-rabbit antibody. Slides were developed with the Dako Liquid 3, ’3-diaminobenzidine tetrahydrochloride (DAB) + Substrate Chromogen System and counterstained with hematoxylin.

The expression levels were scored as proportion of immunopositive staining area (0%, 0; 1–25%, 1; 26–50%, 2; 51–75%, 3; 76%–100%, 4 ) multiplied by intensity of staining (0, negative; 1, weak; 2, moderate; 3, intense). The scores were independently rendered by two pathologists (Dr. Yang YF and Dr. Zhang MF). The median IHC score was chosen as the cut-off value for defining high and low expression.

### Colony formation

Cells were plated into 6-well plates at a density of 500 cells per well. After 10 days of incubation in complete culture medium, the cells colonies were fixed and stained with crystal violet solution (0.1% crystal violet) for 10 min, washed with PBS, and counted.

### Transwell assay

About 3 × 10^4^ cells were cultured in the upper compartment of a Transwell chamber (Corning; 24-well insert, pore size: 8 μm) with FBS-free DMEM. The lower chamber was filled with 15% FBS as a chemoattractant and incubated for 36 h for the migration assay. The cells on the upper surface of the membrane were removed, and the cells on the lower surface were fixed and stained with 0.1% crystal violet. Five visual fields of each insert were randomly chosen and counted under a light microscope.

### Luciferase reporter assay

For the luciferase reporter assay, Bel-7404 cells were co-transfected with FoxM1 overexpression vector, siRNAs or the negative control and 500 ng of psiCHECK-2-PBK-3′-UTR reporter. Cells were collected 36 h after transfection and analysed with the Dual-Luciferase Reporter Assay System (Promega, CA, USA). Luciferase activity was detected by the GloMax fluorescence reader (Promega). The psiCHECK-2 vector that provided the constitutive expression of Renilla luciferase was co-transfected as an internal control.

### Animal model

Male BALB/c-nude mice (4-weeks age, six mice per group) were subcutaneously injected with 1 × 10^7^ cells with PBK overexpression or depletion into the left flanks. Tumors formed by HCC cells were measured with calipers and calculated with the formula: Volume (mm^3^) = [width2 (mm^2^) × length (mm)]/2. At day 28, tumors were dissected and weighed. For metastasis model, approximately 5 × 10^5^ cells were injected via the tail vein. After 6 weeks, mice were sacrificed. The lungs were fixed in 4% paraformaldehyde and stained with hematoxylin and eosin (HE). Lung metastasis was counted and quantified in random selection of high-power fields. All animal studies were approved by the Medical Experimental Animal Care Commission of Sun Yat-sen University Cancer Center.

### Statistical analysis

Data from three separate experiments are presented as mean ± SED. The Student's *t-test* was used for comparisons between groups unless otherwise noted. Kaplan–Meier analyses were used for survival analysis. All data were presented as mean ± SEM of three independent experiments. Differences were considered significant for *P*-values less than 0.05.

## References

[1] Torre LA, Bray F, Siegel RL, Ferlay J, Lortet-Tieulent J, Jemal A (2015). Global cancer statistics, 2012. CA Cancer J Clin.

[2] Njei B, Rotman Y, Ditah I, Lim JK (2015). Emerging trends in hepatocellular carcinoma incidence and mortality. Hepatology.

[3] Chen CL, Pan QZ, Zhao JJ, Wang Y, Li YQ, Wang QJ, Pan K, Weng DS, Jiang SS, Tang Y, Zhang XF, Zhang HX, Zhou ZQ (2016). PD-L1 expression as a predictive biomarker for cytokine-induced killer cell immunotherapy in patients with hepatocellular carcinoma. Oncoimmunology.

[4] Luo CP, Mo HY, Wu LL, Ma Y, Peng NF (2016). Soluble PD-L1 and prognosis of patients with hepatocellular carcinoma. Eur J Cancer.

[5] Boutros T, Chevet E, Metrakos P (2008). Mitogen-activated protein (MAP) kinase/MAP kinase phosphatase regulation: roles in cell growth, death, and cancer. Pharmacol Rev.

[6] Xu Y, Li N, Xiang R, Sun P (2014). Emerging roles of the p38 MAPK and PI3K/AKT/mTOR pathways in oncogene-induced senescence. Trends Biochem Sci.

[7] Dougherty JD, Garcia AD, Nakano I, Livingstone M, Norris B, Polakiewicz R, Wexler EM, Sofroniew MV, Kornblum HI, Geschwind DH (2005). PBK/TOPK, a proliferating neural progenitor-specific mitogen-activated protein kinase kinase. J Neurosci.

[8] Gaudet S, Branton D, Lue RA (2000). Characterization of PDZ-binding kinase, a mitotic kinase. Proc Natl Acad Sci U S A.

[9] Park JH, Lin ML, Nishidate T, Nakamura Y, Katagiri T (2006). PDZ-binding kinase/T-LAK cell-originated protein kinase, a putative cancer/testis antigen with an oncogenic activity in breast cancer. Cancer Res.

[10] Hu F, Gartenhaus RB, Eichberg D, Liu Z, Fang HB, Rapoport AP (2010). PBK/TOPK interacts with the DBD domain of tumor suppressor p53 and modulates expression of transcriptional targets including p21. Oncogene.

[11] Shih MC, Chen JY, Wu YC, Jan YH, Yang BM, Lu PJ, Cheng HC, Huang MS, Yang CJ, Hsiao M, Lai JM (2012). TOPK/PBK promotes cell migration via modulation of the PI3K/PTEN/AKT pathway and is associated with poor prognosis in lung cancer. Oncogene.

[12] Lei B, Qi W, Zhao Y, Li Y, Liu S, Xu X, Zhi C, Wan L, Shen H (2015). PBK/TOPK expression correlates with mutant p53 and affects patients’ prognosis and cell proliferation and viability in lung adenocarcinoma. Hum Pathol.

[13] Ohashi T, Komatsu S, Ichikawa D, Miyamae M, Okajima W, Imamura T, Kiuchi J, Kosuga T, Konishi H, Shiozaki A, Fujiwara H, Okamoto K, Tsuda H (2016). Overexpression of PBK/TOPK relates to tumour malignant potential and poor outcome of gastric carcinoma. Br J Cancer.

[14] Wang MY, Lin ZR, Cao Y, Zheng LS, Peng LX, Sun R, Meng DF, Xie P, Yang JP, Cao L, Xu L, Huang BJ, Qian CN (2016). PDZ binding kinase (PBK) is a theranostic target for nasopharyngeal carcinoma: driving tumor growth via ROS signaling and correlating with patient survival. Oncotarget.

[15] Zhang CZ, Cao Y, Fu J, Yun JP, Zhang MF (2016). miR-634 exhibits anti-tumor activities toward hepatocellular carcinoma via Rab1A and DHX33. Mol Oncol.

[16] Ohashi T, Komatsu S, Ichikawa D, Miyamae M, Okajima W, Imamura T, Kiuchi J, Nishibeppu K, Kosuga T, Konishi H, Shiozaki A, Fujiwara H, Okamoto K (2016). Overexpression of PBK/TOPK Contributes to Tumor Development and Poor Outcome of Esophageal Squamous Cell Carcinoma. Anticancer Res.

[17] Kwon CH, Park HJ, Choi YR, Kim A, Kim HW, Choi JH, Hwang CS, Lee SJ, Choi CI, Jeon TY, Kim DH, Kim GH, do Y Park (2016). PSMB8 and PBK as potential gastric cancer subtype-specific biomarkers associated with prognosis. Oncotarget.

[18] Chen JH, Liang YX, He HC, Chen JY, Lu JM, Chen G, Lin ZY, Fu X, Ling XH, Han ZD, Jiang FN, Zhong WD (2015). Overexpression of PDZ-binding kinase confers malignant phenotype in prostate cancer via the regulation of E2F1. Int J Biol Macromol.

[19] Kim DJ, Li Y, Reddy K, Lee MH, Kim MO, Cho YY, Lee SY, Kim JE, Bode AM, Dong Z (2012). Novel TOPK inhibitor HI-TOPK-032 effectively suppresses colon cancer growth. Cancer Res.

[20] Joel M, Mughal AA, Grieg Z, Murrell W, Palmero S, Mikkelsen B, Fjerdingstad HB, Sandberg CJ, Behnan J, Glover JC, Langmoen IA, Stangeland B (2015). Targeting PBK/TOPK decreases growth and survival of glioma initiating cells *in vitro* and attenuates tumor growth *in vivo*. Mol Cancer.

[21] Song X, Yin S, Zhang E, Fan L, Ye M, Zhang Y, Hu H (2016). Glycycoumarin exerts anti-liver cancer activity by directly targeting T-LAK cell-originated protein kinase. Oncotarget.

[22] Thornton TM, Pedraza-Alva G, Deng B, Wood CD, Aronshtam A, Clements JL, Sabio G, Davis RJ, Matthews DE, Doble B, Rincon M (2008). Phosphorylation by p38 MAPK as an alternative pathway for GSK3beta inactivation. Science.

[23] Gu Y, Wang Q, Guo K, Qin W, Liao W, Wang S, Ding Y, Lin J (2016). TUSC3 promotes colorectal cancer progression and epithelial-mesenchymal transition (EMT) through WNT/beta-catenin and MAPK signalling. J Pathol.

[24] Qu K, Xu X, Liu C, Wu Q, Wei J, Meng F, Zhou L, Wang Z, Lei L, Liu P (2013). Negative regulation of transcription factor FoxM1 by p53 enhances oxaliplatin-induced senescence in hepatocellular carcinoma. Cancer Lett.

[25] Xia L, Huang W, Tian D, Zhu H, Zhang Y, Hu H, Fan D, Nie Y, Wu K (2012). Upregulated FoxM1 expression induced by hepatitis B virus X protein promotes tumor metastasis and indicates poor prognosis in hepatitis B virus-related hepatocellular carcinoma. J Hepatol.

[26] Hu F, Gartenhaus RB, Zhao XF, Fang HB, Minkove S, Poss DE, Rapoport AP (2013). c-Myc and E2F1 drive PBK/TOPK expression in high-grade malignant lymphomas. Leuk Res.

[27] Nandi AK, Rapoport AP (2006). Expression of PDZ-binding kinase (PBK) is regulated by cell cycle-specific transcription factors E2F and CREB/ATF. Leuk Res.

[28] Millour J, de Olano N, Horimoto Y, Monteiro LJ, Langer JK, Aligue R, Hajji N, Lam EW (2011). ATM and p53 regulate FOXM1 expression via E2F in breast cancer epirubicin treatment and resistance. Mol Cancer Ther.

[29] Ho C, Wang C, Mattu S, Destefanis G, Ladu S, Delogu S, Armbruster J, Fan L, Lee SA, Jiang L, Dombrowski F, Evert M, Chen X (2012). AKT (v-akt murine thymoma viral oncogene homolog 1) and N-Ras (neuroblastoma ras viral oncogene homolog) coactivation in the mouse liver promotes rapid carcinogenesis by way of mTOR (mammalian target of rapamycin complex 1), FOXM1 (forkhead box M1)/SKP2, and c-Myc pathways. Hepatology.

